# High Prevalence of Integrative and Conjugative Elements Encoding Transcription Activator-Like Effector Repeats in *Mycoplasma hominis*

**DOI:** 10.3389/fmicb.2019.02385

**Published:** 2019-10-18

**Authors:** Alexandra Meygret, Olivia Peuchant, Emilie Dordet-Frisoni, Pascal Sirand-Pugnet, Christine Citti, Cécile Bébéar, Laure Béven, Sabine Pereyre

**Affiliations:** ^1^USC EA 3671 Mycoplasmal and Chlamydial Infections in Humans, University of Bordeaux, Bordeaux, France; ^2^INRA, USC-EA 3671 Mycoplasmal and Chlamydial Infections in Humans, University of Bordeaux, Bordeaux, France; ^3^Department of Bacteriology, French National Reference Center for Bacterial STI, CHU Bordeaux, Bordeaux, France; ^4^IHAP, INRA, Ecole Nationale Vétérinaire de Toulouse, Université de Toulouse, Toulouse, France; ^5^UMR 1332, BFP, University of Bordeaux, Bordeaux, France; ^6^INRA, UMR 1332, BFP, Bordeaux, France

**Keywords:** *Mycoplasma hominis*, integrative and conjugative element, transcription activator-like effector, horizontal gene transfer, *tet*(M)

## Abstract

Integrative and conjugative elements (ICEs) are modular mobile genetic elements that can disseminate through excision, circularization, and transfer. Mycoplasma ICEs have recently been found distributed among some mycoplasma species and there is accumulating evidence that they play a pivotal role in horizontal gene transfers. The occurrence of ICEs has not been documented in *Mycoplasma hominis*, a human urogenital pathogen responsible for urogenital infections, neonatal infections and extragenital infections. In this study, we searched for, characterized, and compared ICEs by genome analyses of 12 strains of *M. hominis*. ICEs of 27–30 kb were found in one or two copies in seven of the 12 *M. hominis* strains sequenced. Only five of these ICEs seemed to be functional, as assessed by detection of circular forms of extrachromosomal ICE. Moreover, the prevalence of ICEs in *M. hominis* was estimated to be 45% in a collection of 120 clinical isolates of *M. hominis*, including 27 tetracycline-resistant *tet*(M)-positive isolates. The proportion of ICEs was not higher in isolates carrying the *tet*(M) gene, suggesting that ICEs are not involved in tetracycline resistance. Notably, all *M. hominis* ICEs had a very similar structure, consisting of a 4.0–5.1 kb unusual module composed of five to six juxtaposed CDSs. All the genes forming this module were specific to *M. hominis* ICEs as they had no homologs in other mycoplasma ICEs. In each *M. hominis* ICE, one to three CDSs encode proteins that share common structural features with transcription activator-like (TAL) effectors involved in polynucleotide recognition and signal transduction in symbiotic plant pathogen bacteria. The conserved and specific structure of *M. hominis* ICEs and the high prevalence in clinical strains suggest that these ICEs may confer a selective advantage for the physiology or pathogenicity of this human pathogenic bacterium. These data open the way for further studies aiming at unraveling horizontal gene transfers and virulence factors in *M. hominis*.

## Introduction

Mycoplasma species represent a large group of wall-less bacteria derived from a common ancestor to Gram-positive bacteria, such as *Clostridia* (Woese et al., 1980). Their evolution has been marked by loss of genetic material, resulting in some species, such as *Mycoplasma genitalium*, which are considered good natural representatives of minimal cells (Glass et al., 2006) because of their reduced genome. Genome down-sizing was not the sole force operating during mycoplasma evolution; it was recently shown that mycoplasmas sharing the same natural niche have exchanged significant amounts of their genome through horizontal gene transfer (HGT) (Sirand-Pugnet et al., 2007; Pereyre et al., 2009). A few mobile genetic elements (MGE), including insertion sequences, phages, plasmids, and integrative and conjugative elements (ICEs), have been described in mycoplasmas (Breton et al., 2012; Marenda, 2014) and may be involved in such genetic transfer.

Over the last several decades, there has been increasing appreciation of the role of ICEs in HGT (Burrus and Waldor, 2004; Delavat et al., 2017). Their importance in bacterial evolution is underlined by their wide distribution across bacteria. These genetic elements are defined by two features: their integration in the host chromosome and their capacity to encode a type IV secretion system that mediates their transfer from the donor cell to the recipient cell via conjugation (Johnson and Grossman, 2015). Mycoplasma ICEs (MICEs) are emerging as pivotal factors in HGT of large DNA fragments in many mycoplasmas species (Citti et al., 2018; Faucher et al., 2019). The first reported MICE was ICEF, a genetic element of 23 kb occurring in four copies in *Mycoplasma fermentans* PG18 (Calcutt et al., 2002), a human mycoplasma species of the Hominis phylogenetic group. A second MICE of 27 kb, designated as ICEA, was identified in three copies in *Mycoplasma agalactiae* strain 5632 (Marenda et al., 2006), a ruminant mycoplasma belonging to the same phylogenetic group. Both MICEs are composed of about 20 structural genes, of which 12 are homologous. Similar MICEs have also been detected in other animal mycoplasma species, such as *Mycoplasma bovis* (Wise et al., 2011), *Mycoplasma mycoides* subsp. *capri*, and *Mycoplasma capricolum* subsp. *capricolum* (Thiaucourt et al., 2011). These self-transmissible elements belong to a new clade of the mutator-like superfamily and rely on a DDE recombinase for their mobility (Dordet Frisoni et al., 2013; Guerillot et al., 2014). In 2015, a comparative study including MICEs found, in several ruminant mycoplasmas species, a conserved, minimal MICE backbone composed of four coding sequences (CDSs) predicted to be essential for MICE self-dissemination across cells (Tardy et al., 2015): CDS1, of unknown function, CDS22 encoding a DDE recombinase, and CDS5 and CDS17 encoding TraG/VirD4 and TraE/VirB4 homologs, respectively, which are predicted to be two essential components of the type IV secretion system required for DNA mobility (Citti et al., 2018). The detection of MICEs in several mycoplasma species of the Hominis phylogenetic group, which includes the human *M. fermentans* species, raised the question of whether they also occur in the human *M. hominis* species.

*Mycoplasma hominis* is present as a commensal in the lower urogenital tract but can be responsible for urogenital infections, neonatal infections, and extragenital infections in immunocompromised patients (Waites and Talkington, 2005; Waites et al., 2009). Recent evidence of HGT with *Ureaplasma parvum*, a species sharing the same urogenital habitat, has been reported despite the absence of plasmids or transposons in the *M. hominis* type strain PG21 (Pereyre et al., 2009). The MGEs have rarely been described in other *M. hominis* isolates. To date, the transposon Tn*916* (Roberts et al., 1985; Mardassi et al., 2012), other transposons carrying the *tet*(M) gene (Allen-Daniels et al., 2015; Calcutt and Foecking, 2015c) and prophages (Allen-Daniels et al., 2015; Calcutt and Foecking, 2015b) have been reported in *M. hominis* isolates. In addition, no MICE similar to those described in *M. fermentans* or ruminant mycoplasmas have been explored in *M. hominis*. In the present study, detection and characterization of *M. hominis* ICEs was performed in one reference strain and 11 clinical isolates by whole-genome sequencing and analyses. Common and specific *M. hominis* ICE (ICEHo) determinants were further defined by comparative genomics. In addition, the prevalence of ICEHos in *M. hominis* species was estimated by screening a collection of 120 clinical isolates.

## Materials and Methods

### Mycoplasma Strains

The genomes of reference strain M132 and 11 French clinical isolates of *M. hominis* were sequenced. The 11 clinical isolates were part of the isolate collection of the French National Reference Center for sexually transmitted bacterial infections and have been preserved at the Centre de Ressource Biologique-Bordeaux Biothèque Santé (CRB-BBS) of Bordeaux University Hospital under the collection number BB-0033-00094 and authorization AC-2014-2166 from the French Ministry of Higher Education and Research with no information regarding the patient identity from whom the isolate had been grown. These strains were chosen among a total of about 500 *M. hominis* isolates to ensure diversity in terms of sampling origin, multi-locus variable-number tandem-repeat (VNTR) analysis (MLVA) types and years of isolation (Table 1).

**TABLE 1:** *M. hominis* strains used for genome sequencing.

**Strain**	**Clinical context**	**Nature of the sample**	**Geographic origin**	**Year of isolation**	**MLVA type (Ferandon et al., 2013)**
M132			Reference strain		10
35	Neonatal fever	Blood culture	Bordeaux	1989	8
331	Psoriatic arthritis	Joint fluid	Bordeaux	1991	15
2674	Mediastinitis	Mediastinal suppuration	Paris	1998	10
3299	Osteoarthritis	Joint fluid	Bordeaux	2002	36
3631	Endometritis	Amniotic liquid	Bordeaux	2004	4
4016	Endometritis	Endometrial biopsy	Lannion	2005	8
4235	Salpingitis	Peritoneal fluid	Bordeaux	2006	10
4788	Vaginosis	Vaginal secretions	Bordeaux	2008	10
5012	Commensal	Semen	Bordeaux	2009	12
5060	Vaginosis	Vaginal secretions	Bordeaux	2009	8
5096	Maternal-fetal infection	Placenta	Bordeaux	2009	15

To determine the prevalence of ICE in *M. hominis* species, 120 clinical isolates randomly selected from among 183 isolates collected in a previous study (performed between 1 January 2010 and 31 December 2015 at Bordeaux University Hospital, France) were used (Meygret et al., 2018). The sources of the samples were diverse and included 75 (62.5%) urogenital, 25 (20.8%) placental, 2 (1.7%) lower respiratory, 2 (1.7%) upper respiratory samples, 5 (4.2%) puncture liquids from different origin (1 cerebrospinal fluid, 1 puncture liquid from the pouch of Douglas, and 3 puncture liquids of unknown origin), as well as 11 (9.2%) samples of unknown origin. Among the 120 clinical isolates, 27 isolates harbored the *tet*(M) gene (Meygret et al., 2018).

### DNA Extraction

The extraction of genomic DNA was performed using Nucleobond^®^ AXG 20 columns with a Nucleobond Buffer set III kit (Macherey Nagel, Hoerdt, France) according to the manufacturer’s instructions for Illumina sequencing, and using an increased initial culture volume of 10 mL for Oxford Nanopore sequencing to increase the DNA concentration. A NucleoSpin^®^ Tissue kit (Macherey Nagel) was used for DNA extraction for conventional PCR.

### Sequencing and Assembly

Mate pair reads were generated with Illumina GAIIx technology for four strains, i.e., *M. hominis* 331, 3631, 4016, and 5012 (6,000X average coverage), and with the MiSeq system for the other seven strains (775X average coverage; Illumina, San Diego, CA, United States). Paired-end reads were also generated with Illumina GAIIx technology (1,700X average coverage) for all strains. Contigs were obtained using two *de novo* assembly tools, ABySS (Simpson et al., 2009) (version 1.3.5) and CLC assembly cell (version 4.2.0)^1^.

Strains 4788, 4235, and 35 were also sequenced with Oxford Nanopore technology using a GridIon apparatus. To generate the assemblies, a hybrid approach consisting of the assembly of long reads only and polishing of the resulting contigs with short reads was implemented. Long reads with Phred score <Q9 and minimum length <1,000 bp were removed with Filtlong v0.1.1^2^. The theoretical depth of remaining reads for each seed was greater than 250X. Assembly was performed with Canu1.7 (Koren et al., 2017) using the default parameters and an expected genome size of 800 kb. Before polishing draft assemblies with Pilon 1.22 (Walker et al., 2014), Illumina reads were trimmed using Trimmomatic V0.36 (Bolger et al., 2014). Sequences were annotated with Prokka v1.12 (Seemann, 2014), using the previously published annotation of the *M. hominis* PG21 strain genome as a reference (Pereyre et al., 2009).

### Gap Closures, Genome Analysis, and Comparisons

Detection of contigs harboring ICE-related CDSs was performed by comparison with the previously reported genome sequences of *M. fermentans* PG18 (GenBank accession number: AP009608), *M. agalactiae* 5632 (FP671138), *M. bovis* PG45 (CP002188), *M. bovis* Hubei (CP002513), and *M. mycoides* subsp. *capri* GM12 (CP001668) using MolliGen (Barre et al., 2004). The characterization of ICEHo 4788, carried by a single contig, allowed detection of contigs carrying fragments of ICEHo in the other fully sequenced strains. Gap closure between contigs carrying ICE sequences was performed by PCR and Sanger sequencing. ICE maps were drawn using Clone Manager 9 Basic Edition (Scientific & Educational Software, Denver, CO, United States).

The ICE-related CDSs were analyzed using BLAST^3^, InterPro^4^, MolliGen^5^, Artemis Comparison Tool^6^, EMBOSS Needle^7^, TMHMM Server v.2.0^8^, MEME analysis^9^, RADAR^10^, SPINE-D^11^, PHYRE2^12^, IsUnstruct v2.02^13^, and PONDR^®^.^14^

### Southern Blotting Analyses

Southern blotting analyses were performed as described previously (Marenda et al., 2006). Briefly, 1 μg of genomic DNA was separately digested with *Hin*dIII and *Eco*RV. Membranes were hybridized with digoxigenin-labeled (Roche, Mannheim, Germany) CDS1-specific or CDS22-specific probes generated by PCR (Table 2).

**TABLE 2:** Primers used to detect the four ICE backbone CDSs (CDS1, CDS5, CDS17, CDS22) and the specific *M. hominis* ICE module.

**Forward primers**	**Sequences of forward primers (5′-3′)**	**Reverse primers**	**Sequences of reverse primers (5′-3′)**	**Size of PCR product (bp)**
CDS1-F cons	TTGAAATAAARGTTTTAGATGRTGAG	CDS1-R cons	CAAACATTTGCAAATATYTAACTCCA	290
CDS5-F cons	TGAGATTTAGCGGTTGATGGYG	CDS5-R cons	TACTTGRGCTGTTCCTTCYC	771
CDS17-F cons	TRAAGCCAAAMAAGCAAAGC	CDS17-R cons	MGCCATTGTYGCTTGTTCATC	581
CDS22-F cons	TTCAGCTAGAAAATGAYGATGC	CDS22-R cons	RCTRGTTTTAGTGGYTAAGA	146
MhoF cons	TCATCCCATACCCCCTCK	MhoR-cons	TTAAAACGACCGCCTTGGTTG	4000 to 5100

### Detection of ICE-Related CDS and ICE Extrachromosomal Forms

Detection of the four conserved ICE genes (CDS1, CDS5, CDS17, and CDS22) was performed by PCR (Table 2). The primers for detection of the specific *M. hominis* ICE module were designed to correspond to the flanking CDSs, i.e., CDS11 for the forward primer and CDS14 for the reverse primer. The extrachromosomal ICEHo circular forms (cICEHos) were detected using outward facing primers (Supplementary Table S1).

PCR for detection of the four conserved CDSs was carried out in a volume of 50 μL using GoTaq G2 hot start polymerase (Promega, Madison, WI, United States). Thermal cycling reactions consisted of an initial denaturation step of 15 min at 95°C followed by 30 cycles of denaturation for 1 min at 95°C, annealing for 1 min at 56°C, and extension for 2 min at 72°C, with a final extension for 5 min at 72°C.

PCR for detection of the specific *M. hominis* ICE modules was carried out in a volume of 50 μL using the Expand Long Template PCR system (Roche). Thermal cycling reactions consisted of an initial denaturation step for 2 min at 95°C followed by 15 cycles of denaturation for 10 s at 95°C, annealing for 30 s at 57°C, and extension for 2 min at 68°C, and 20 cycles of denaturation for 15 s at 95°C, annealing for 30 s at 57°C, and extension for 2 min + 20 s for each successive cycle at 68°C, with a final extension for 7 min at 68°C.

All PCR products were sent to Eurofins Genomics (Ebersberg, Germany) for sequencing. Sequencing data were analyzed using BioEdit^§^ 7.2.5 software (Isis Pharmaceuticals, Carlsbad, CA, United States).

### Statistical Analysis

The chi-squared test was used to compare the prevalence of ICEHos between isolates carrying the *tet*(M) gene and those without the *tet*(M). *P* < 0.05 was considered significant.

### Data Availability

Illumina reads have been deposited in SRA under BioProject PRJNA493181. Strain 4788, 4235 and 35 genome assemblies have been submitted to GenBank under the accession numbers CP035542, CP038014, and CP035543, respectively.

## Results

### Detection of MICEs in *M. hominis*

We first searched for the minimal ICE backbone composed of CDS1, CDS5, CDS17, and CDS22 using the Molligen database in the 12 fully sequenced *M. hominis* genomes. Three isolates, i.e., 331, 3299, and 5096, had none of the four CDSs. Two isolates, i.e., 2674 and 5060, had three CDSs (CDS1, CDS5, and CDS22), but they occurred at different loci suggesting that they were vestigial ICE remnants. The minimal ICE backbone was detected in the remaining seven isolates, suggesting the presence of complete ICEs. A full ICE, designated as ICEHo 4788, was identified in isolate 4788 and was carried by a single contig. Its organization and composition were defined and ICEHo 4788 was then used as a reference to analyze and assemble other isolates, in which ICE elements were distributed over several contigs.

### *M. hominis* 4788 ICE

**FIGURE 1:**
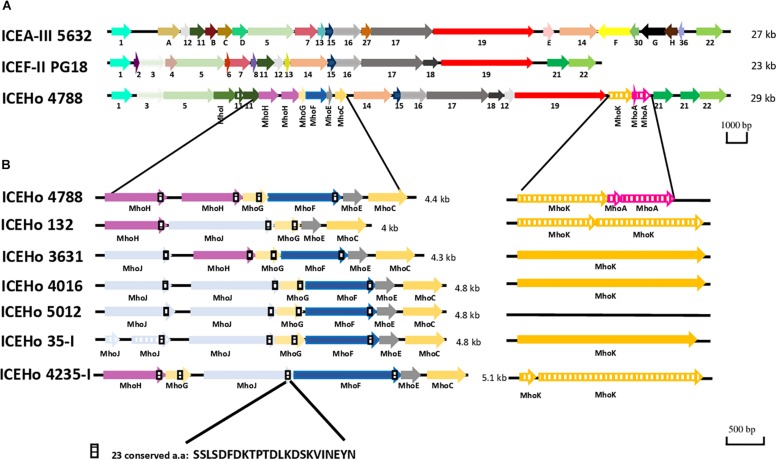
Genetic organization of integrative and conjugative elements (ICEs) from *M. hominis*, *M. agalactiae*, and *M. fermentans*. **(A)** Comparison of ICEs present in *M. hominis* 4788 (ICEHo 4788), *M. agalactiae* 5632 (ICEA-III), and *M. fermentans* PG18 (ICEF-II). **(B)** Genetic organization of the specific *M. hominis* ICE module in the ICEHos of the seven sequenced *M. hominis* strains harboring complete ICEHos. ICEs are designated according to their originally proposed names. *M. hominis* ICEs are designated with same number as the *M. hominis* strain from which they are originated from (e.g., ICEHo 4788 is from *M*. *hominis* 4788 strain). Arrows represent coding sequences (CDSs), hatched arrows represent pseudogenes. Homologous CDSs are identified by the same color and same number or letter underneath, using previously described nomenclature. ICEHo 4788 CDSs that had no homology with CDSs reported previously in mycoplasma ICEs (MICEs) were designated as Mho plus a letter (MhoA to MhoK).

ICEHo 4788 is a large genetic element of 29,180 bp, accounting for 4.1% of the *M. hominis* 4788 genome (712,285 bp). Its GC content was 27.31%, which was not significantly different from the GC content of 26.98% in the *M. hominis* 4788 genome (*P* = 0.622, χ^2^ test). Comparative analysis of ICEHo 4788 CDS synteny and amino acid (aa) identity and similarity with ICEF-II from the *M. fermentans* PG18 strain (Calcutt et al., 2002) and ICEA-III from the *M. agalactiae* 5632 strain (Marenda et al., 2006) was performed (Figure 1A, Table 3, and Supplementary Table S2). ICEHo 4788 contained 25 CDSs, including 13 distinct CDSs homologous to ICEF-II CDSs, which were further designated using the same nomenclature (Calcutt et al., 2002). The synteny was overall conserved between ICEHo 4788 and ICEF-II but CDS12 was located at different positions—downstream of CDS18 in ICEHo 4788 and downstream of CDS11 in ICEF-II (Figure 1A). When compared to ICEA-III (Marenda et al., 2006), only 10 distinct homologous CDSs were found in ICEHo 4788 (Figure 1, Table 3, and Supplementary Table S2). Overall, the percentages of similarity and identity between CDSs of ICEHo 4788 and ICEA-III were lower than those between ICEHo 4788 and ICEF-II (Table 3 and Supplementary Table S2). Using TMHMM and InterPro softwares, six CDSs (CDS5, CDS14, CDS15, CDS16, CDS17, and CDS19) were shown in ICEHo 4788 to harbor transmembrane domains in comparison to eight CDSs previously described in ICEF-II (CDS4, CDS5, CDS7, CDS14, CDS15, CDS16, CDS17, CDS19) (Calcutt et al., 2002) and seven in ICEA-III (CDS5, CDS7, CDS14, CDS15, CDS16, CDS17, CDS19) (Marenda et al., 2006). Marked differences were observed with *M. fermentans*, as six ICEF-II CDS homologs were lacking in ICEHo 4788 (CDS2, CDS4, CDS6, CDS7, CDS8, CDS13) and eight ICEHo 4788 CDSs had no homology with ICEF-II CDSs, nor with CDSs described previously in MICEs. These specific CDSs were designated as Mho plus a letter (MhoA to MhoK). Their best NCBI Blast hits retrieved mainly hypothetical proteins and methyltransferases from bacteria belonging to the same urogenital environment, such as *Ureaplasma parvum*, *Candidatus* Mycoplasma girerdii, and *Finegoldia magna* (Table 4).

**TABLE 3:** Comparison of CDSs from *M. hominis* ICEHo 4788 with CDSs from *M. fermentans* PG18 ICEF-II and *M. agalactiae* 5632 ICEA-III.

***M. fermentans* PG18 ICEF-II**						***M. agalactiae* 5632 ICEA-III**					
			
**ICEHo 4788**		**Size (aa) of PG18 ICEF-II CDSs**	**Length of alignment ICEHo 4788 *vs.* ICEF-II**	**% identity (Nb identical aa/length of alignment)**	**% similarity (Nb similar aa/length of alignment)**	**% gaps (Nb gaps/length of alignment)**	**Size (aa) of 5632 ICEA-III CDSs**	**Length of alignment ICEHo 4788 *vs.* ICEA 5632-III**	**% identity (Nb identical aa/length of alignment)**	**% similarity (Nb similar aa/length of alignment)**	**% gaps (Nb gaps/length of alignment)**

**CDS**	**Size (aa)**										
CDS1	263	263	290	32.8 (95/290)	46.9 (136/290)	18.6 (54/290)	264	300	25.3 (76/300)	44.3 (133/300)	24.3 (73/300)
CDS3	413	400	422	37.2 (157/422)	58.3 (246/422)	7.3 (31/422)	None	None	None	None	None
CDS5	766	740	811	35.8 (290/811)	54.3 (440/811)	14.3 (116/811)	670	813	32.7 (266/813)	49.8 (405/813)	23.4 (190/813)
MhoI	335	None	None	None	None	None	None	None	None	None	None
CDS11	252	238	260	30.4 (79/260)	50.4 (131/260)	11.5 (30/260)	221	302	14.9 (45/302)	27.8 (84/302)	43.4 (131/302)
MhoH	266	None	None	None	None	None	None	None	None	None	None
MhoG	102	None	None	None	None	None	None	None	None	None	None
MhoF	328	None	None	None	None	None	None	None	None	None	None
MhoE	63	None	None	None	None	None	None	None	None	None	None
MhoC	169	None	None	None	None	None	None	None	None	None	None
CDS14	562	552	611	24.4 (147/611)	43.4 (261/611)	21.1 (129/611)	525	622	22.8 (142/622)	40.8 (254/622)	25.2 (157/622)
CDS15	120	95	126	25.4 (32/126)	39.7 (50/126)	29.4 (37/126)	121	126	29.4 (37/126)	50.8 (64/126)	8.7 (11/126)
CDS16	400	396	416	27.4 (114/416)	47.1 (196/416)	8.7 (36/416)	357	434	22.4 (97/434)	38.9 (169/434)	25.6 (111/434)
CDS17	927	937	955	39.8 (380/955)	59.1 (564/955)	4.8 (46/955)	928	950	40.2 (382/950)	60.0 (570/950)	4.7 (45/950)
CDS18	260	227	281	24.9 (70/281)	45.6 (128/281)	26.7 (75/281)	None	None	None	None	None
CDS12	130	183	195	26.2 (51/195)	39.0 (76/195)	39.5 (77/195)	134	142	24.6 (35/142)	50.0 (71/142)	14.1 (20/142)
CDS19	1434	1424	1566	28.0 (439/1566)	45.0 (704/1566)	17.5 (274/1566)	1517	1720	24.8 (426/1720)	39.1 (673/1720)	28.4 (489/1720)
MhoK	363	None	None	None	None	None	None	None	None	None	None
MhoA	232	None	None	None	None	None	None	None	None	None	None
CDS21	286	313	325	56.6 (184/325)	65.2 (212/325)	15.7 (51/325)	None	None	None	None	None
CDS22	396	390	427	23.2 (99/427)	36.3 (155/427)	34 (145/427)	378	412	23.8 (98/412)	36.4 (150/412)	30.8 (127/412)
MhoJ*^*a*^*	464	None	None	None	None	None	None	None	None	None	None

*Percentages of amino acid identity and similarity were determined using EMBOSS Needle. ICEF-II: second ICE of *M. fermentans* PG18 (Calcutt et al., 2002). ICEA 5632-III: third copy of ICE in *M. agalactiae* 5632 (Marenda et al., 2006). *^*a*^*MhoJ is not harbored by *M. hominis* 4788, but is harbored by the six other sequenced *M. hominis* strains harboring ICEs.*

**TABLE 4:** Major features of specific *M. hominis* ICE CDSs.

**Specific *M. hominis* ICE CDSs**	**ICEHo CDS size (aa)**	**NCBI best Blast hit^a^**	**Organism**	***E*-value^a^**	**% coverage^a^**	**% Identity^a^**	**Best Blast hit size (aa)**	**Length of align ment^b^ (aa)**	**% identity vs. best Blast hit^b^**	**% similarity vs. best Blast hit^b^**	**% gaps vs. best Blast hit^b^**
MhoI	335	DNA cytosine methyl transferase	*Ureaplasma parvum*	1e-129	59	92.5	200	335	55.2 (185/335)	56.7 (190/335)	40.3 (135/335)
MhoH	266	Hypothetical protein	*Finegoldia magna*	6e-44	81	46	300	336	33.0 (111/336)	45.8 (154/336)	31.5 (106/336)
MhoG	102	Hypothetical protein	*Ca.* Mycoplasma girerdii	4e-8	35	78	179	190	23.2 (44/190)	32.1 (61/190)	52.1 (99/190)
MhoF	328	Hypothetical protein	*Finegoldia magna*	3e-53	90	41	300	366	34.4 (126/366)	48.9 (179/366)	28.4 (104/366)
MhoE	63	Hypothetical protein	*Mycoplasma fermentans*	7e-13	95	52	60	63	49.2 (31/63)	71.4 (45/63)	4.8 (3/63)
MhoC	169	Phage protein	*Mycoplasma hyorhinis*	3e-53	97	50	223	223	37.2 (83/223)	52.9 (118/223)	24.2 (54/223)
MhoK	363	Site-specific DNA methyl transferase	*Ureaplasma parvum*	0	100	97	363	363	96.7 (351/363)	98.1 (356/363)	0 (0/363)
MhoA	232	Hypothetical protein	*Ureaplasma parvum*	5e-158	99	96	264	264	83.7 (221/264)	86.7 (229/264)	12.1 (32/264)
MhoJ^*c*^	464	Hypothetical protein	*Ca.* Mycoplasma girerdii	4e-43	50	66	179	472	25.6 (121/472)	30.5 (144/472)	63.8 (301/472)

*^*a*^Determined using BLAST. ^*b*^Percentages of amino-acid identity and similarity with the NCBI best BLAST hits were determined using EMBOSS Needle. ^*c*^MhoJ is not harbored by *M. hominis* 4788 but is harbored by the six other sequenced *M. hominis* strains harboring ICEs.*

### Common and Specific Features of *M. hominis* ICEs

In addition to ICEHo 4788, complete ICEs were detected and characterized in six strains (ICEHo 35-I, ICEHo 132, ICEHo 3631, ICEHo 4016, ICEHo 4235-I, and ICEHo 5012) (Supplementary Figure S1). Overall, ICEHos showed a similar structure except for the occurrence of MhoA as a pseudogene, which was only present in ICEHo 4788 (Supplementary Figure S1). All ICEHos contained a 4.0–5.1-kb module composed of five to six juxtaposed CDSs, i.e., MhoJ, MhoH, MhoG, MhoF, MhoE, and MhoC, the synteny of which varied slightly among the strains, as shown in Figure 1B. All of the genes forming this module were specific to *M. hominis* ICEs (see below for detailed description) as they had no homologs in other MICEs.

### *M. hominis* ICEHo May Occur as Multiple Copies

Southern blot hybridization data using labeled CDS1 and CDS22 probes and *Eco*RV-digested chromosomal DNA suggested the presence of a single chromosomal ICEHo in *M. hominis* strains 3631, 132, 4016, 4788, and 5012, and the presence of at least two ICEHo copies in strains 4235 and 35 (Supplementary Figure S2). Southern blot hybridization performed with *Hin*dIII-digested chromosomal DNA yielded the same results (data not shown). In strains 4235 and 35, Illumina sequencing retrieved a single copy of ICEHo due to the limits of the assembly process based on short reads only. To overcome this issue, the genomes of *M. hominis* strains 4788, 4235, and 35 were sequenced using Oxford Nanopore technology that can generate long reads. The combined assembly of Oxford Nanopore and Illumina reads confirmed the presence of a single ICEHo in strain 4788 and revealed the occurrence of two ICEHo 4235 copies in *M. hominis* 4235, namely ICEHo 4235-I of 30.5 kb at position 397,452–428,015 and ICEHo 4235-II at position 778,637–809 029 (GenBank accession number CP038014). The two copies showed 99.0% sequence identity and were in opposite orientation within the genome of *M. hominis* 4235. In *M. hominis* strain 35, the presence of two ICEHo copies, namely ICEHo 35-I of 30.3 kb (position 449,355–479,744) and ICEHo 35-II of 29.1 kb (position 312,315 to 341,498) (GenBank accession number CP035543) was also confirmed. These two copies were in the same orientation within the genome and showed 90.0% sequence identity.

### Extrachromosomal Circular Forms and Sites of Integration of ICEHos

**FIGURE 2:**
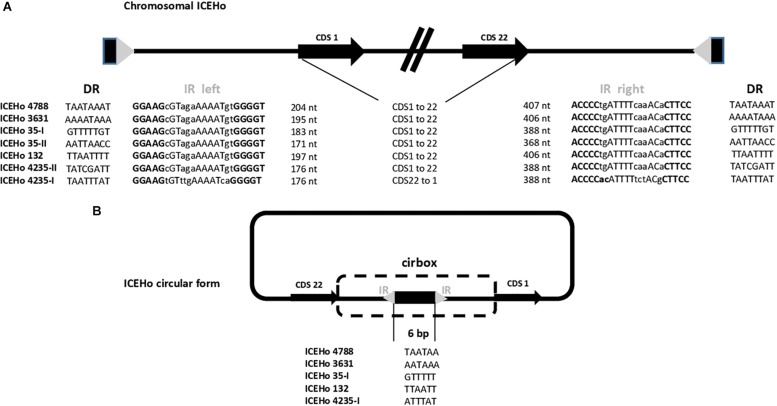
Chromosomal and extrachromosomal circular forms of ICEHo. **(A)** Schematic representation of the chromosomal loci containing ICEHos in the five sequenced *M. hominis* strains harboring ICEHos able to circularize. The chromosomal ICEs possess two imperfect inverted repeats (IRs, represented by a gray triangle) at each end that are flanked by two 8-bp direct repeats (DRs, represented by a black rectangle). **(B)** Extrachromosomal ICEHo circular forms with details of the 6-bp coupling sequences. Excision and circularization from the *M. hominis* donor chromosome resulted in a free circular form, in which the junction contains a 6-bp coupling region. This coupling region corresponds to a juxtaposition of the DRs flanking the ICEHo.

One piece of evidence for ICE functionality is the detection of a circular form due to chromosomal excision. To detect these cICEHos in *M. hominis*, outward facing primers located at both ends of the chromosomal ICEs were designed for all strains (Supplementary Table S1). PCR products were detected for five strains, i.e., *M. hominis* 4788, 3631, 35, 132, and 4235, and the cICEHo junctions previously designated as cirboxes (Tardy et al., 2015) were sequenced (Figure 2A). As shown in Figure 2B, these sequences encompassed the downstream region of CDS22, a non-coding region, an imperfect inverted repeat (IR), a 6-bp coupling sequence corresponding to a juxtaposition of the two 8-bp direct repeats that flank the ICEHo, an imperfect IR, and a non-coding region located upstream of CDS1 (Figure 2A). Cirbox sequences were highly conserved with 92% identity between cirboxes of ICEHo 4788, ICEHo 3631, ICEHo 35-I, ICEHo 132, and ICEHo 4235-I. The length of the non-coding regions between CDS22 and the IR, and between the IR and CDS1 were similar, ranging between 368 and 407 nt and between 171 and 204 nt, respectively. The 6-bp coupling sequences differed between strains (Figure 2B). No cICEHo was detected for ICEHo 4016 or ICEHo 5012, but we noted that these two ICEs had truncated ends (Supplementary Figure S1). Indeed, ICEHo 4016 CDS1 comprised only 171 aa compared to 263 aa for the other ICEHo CDS1 (35% loss of length). ICEHo 5012 CDS22 was comprised of 142 aa and was thus shorter than the other ICEHo CDS22 (396 aa; 36% loss of length). Furthermore, IRs and direct repeats were not found in ICEHo 5012 or ICEHo 4016.

The chromosomal locations of ICEHos are shown in Supplementary Figure S3. The results indicated that ICEHos could insert in either coding or non-coding regions without any target specificity.

### Analysis of the Specific *M. hominis* ICE Module

Further analysis of the specific *M. hominis* ICE module highlighted that in MhoH, MhoG, MhoF, and MhoJ, an absolutely conserved sequence of 23 aa (SSLSDFDKTPTDLKDSKVINEYN) was present within the last 40 residues of the sequences (Figure 1). This conserved sequence was also detected in CDS11 and CDS21, but a Prosite search failed to retrieve functional motifs. The conserved sequence contained a large number of charged amino acids, with five D/E and three K residues, and was also found in a hypothetical protein from *Candidatus* Mycoplasma girerdii (MGM1_3750). The C-terminal part of the proteins containing this conserved motif was predicted to be disordered using SPINE-D, Phyre2, and IsUnstruct v2.02, and was enriched in putative phosphorylation sites (prediction using DEPP^®^ server) (Supplementary Figure S4).

**FIGURE 3:**
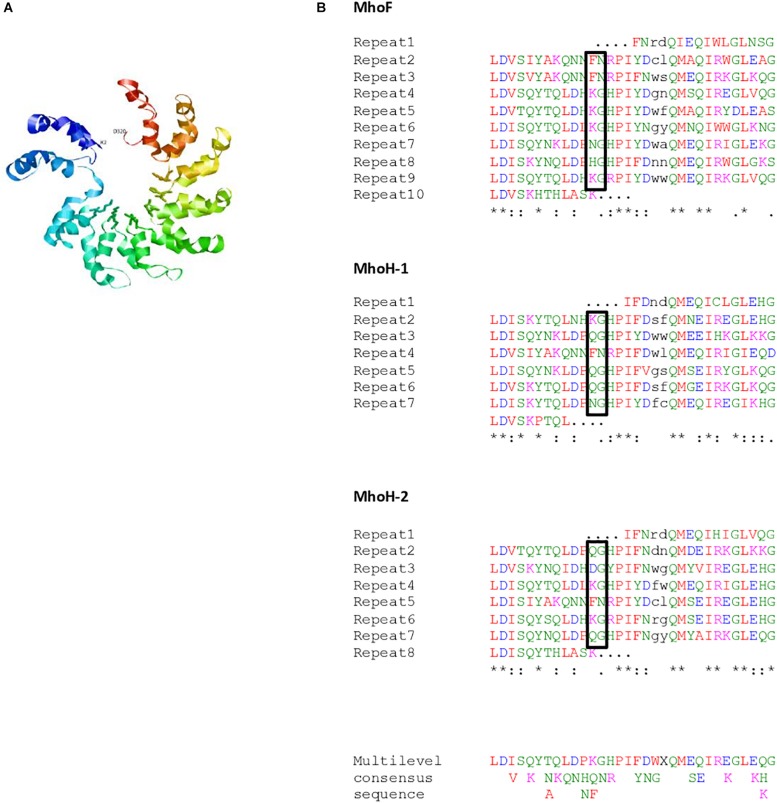
**(A)** Predicted structure for MhoF (residues 2–320) modeled using Phyre2. Variable residues (putative repeat variable di-residue [RVD]) at positions 12 and 13 of the double helix are shown using balls and sticks representation. **(B)** Repeated sequences in MhoF and MhoH sequences. The repeated sequences identified using the RADAR algorithm were further refined using Phyre2 alignments. Repeats were aligned using Clustal Omega. Amino acids (aa) with similar physicochemical properties are shown in the same color: red, small and hydrophobic aa; blue, acidic aa; magenta, basic aa; green, hydroxyl, sulfhydryl, and amine aa. The two residues of the double helix that are not repeated are indicated in black. Most variable di-residues within MhoF repeats, and corresponding di-residues in MhoH, are shown in boxes. ^∗^Conserved residues among repeats for each protein without taking into account the truncated repeats. The multilevel consensus sequence was identified using MEME software.

In the search for additional insights regarding the putative function of ICEHo 4788 proteins, *in silico* analysis using Phyre2 (secondary and tertiary structure elements) and RADAR (direct repeat) algorithms was performed for detailed analysis of the primary protein sequences. A modeled partial structure was obtained using Phyre2 for the 10 proteins listed in Table 5. All proteins for which a structure was obtained, except MhoK, were predicted to share structural similarities to DNA interacting/modifying proteins. Phyre2 analysis of secondary structures in MhoF and MhoH revealed a high content of α-helices, organized in a tight stretch of supersecondary elements consisting of α-helices linked by short loops. Phyre2 3-D structure prediction indicated that both proteins shared common structural features with transcription activator-like (TAL) effectors (Table 5). The highest scoring templates leading to modeling of MhoF and MhoH structures were structures of the TAL effector AvrBs3/PthA for MhoF and the second copy of ICEHo 4788 MhoH (98 and 95% coverage, respectively), and BurrH DNA binding protein for the first copy of MhoH (97% coverage). The modeled structure obtained for MhoF is shown in Figure 3A. Similar to BurrH and in contrast to typical TAL effectors, such as AvrBs3, MhoF and MhoH do not possess any recognizable nuclear localization signal (NLS) sequence, N-terminal type III secretion signal or activation domain at the C-terminus.

**TABLE 5:** Phyre2 models for ICEHo 4788 proteins.

**ICEHo 4788**	**Phyre 2 top hit template**				
	
**CDS**	**Template**	**Chain information**	**Organism**	**% Coverage (number of aa)**	**% Confidence**
CDS3	c4lvjA	MobM relaxase domain	*Streptococcus agalactiae*	31 (126)	97.6
CDS5	d1eçRa	RecA protein like (ATPase domain)	*Escherichia coli*	51 (392)	100
MhoI	d2c7pa1	C5 cytosine DNA methyltransferase DCM	*Haemophilus haemolyticus*	95 (319)	100
MhoH-1	c4cj9A	DNA binding protein BurrH	*Burkholderia rhizoxinica*	97 (262)	100
MhoH-2	c3UGmA	TAL effector PthXo1	*Xanthomonas oryzae* pv. *oryzae*	95 (253)	100
MhoF	c3UGmA	TAL effector PthXo1	*Xanthomonas oryzae* pv. *oryzae*	98 (322)	100
MhoC	c1aqjB	N6 adenine DNA methyltransferase *Taq*I	*Thermus aquaticus*	72 (121)	99.6
CDS17	c4ag5a	Type IV secretory pathway component VirB4	*Thermoanaeobacter pseudethanolicus*	40 (373)	100
CDS12	c1qvcA	Single stranded DNA binding Protein 2	*Escherichia coli*	95 (123)	100
MhoK	d1G60A	*S*-adenosyl-L-methionine-dependent methyltransferase	*Moraxella bovis*	64 (233)	100

*Only models of proteins for which coverage was higher than 120 amino acids, associated with a confidence higher than 95%, are listed.*

Combination of Phyre2 alignments and RADAR results allowed identification of eight 32-residue long repeats in MhoF, and six repeats of the same length in both MhoH sequences in ICEHo 4788 (Figure 3B). In all proteins, the full-length repeats were flanked by two supplementary truncated repeats. In addition, the C-terminal ends of all three proteins were predicted to form a double helix containing two cryptic repeats, which included the 23-aa conserved motif. Alignment of the whole set of 32-residue long repeats found in MhoF and MhoH sequences using Clustal Omega revealed the presence of eight strictly conserved aa (Figure 3B). Following the Phyre2 model (Figure 3A), repeats were associated to form a structure based on successive two-helix bundles. Whereas in typical TAL effectors repeated sequences are almost identical, except at positions 12 and 13 corresponding to the repeat variable di-residue (RVD) motif involved in nucleotide recognition, a high degree of polymorphism of Mho TAL-like repeats was observed. Nonetheless, at positions 12 and 13 of the repeats in MhoF, variable residues (KG, FN, NG, and HG) exposed in interhelical loops were identified as the putative RVD. The other highly variable positions were positions 20 and 21 of each repeat of MhoF, but the corresponding residues were part of the helical structures predicted using Phyre2. The truncated repeats (1st and 10th repeats) in MhoF did not contain a putative RVD. Examination of MhoH sequences suggested that QG may also correspond to an RVD (Figure 3B).

### Prevalence of ICEHo in a Collection of 120 *M. hominis* Clinical Isolates

To define the prevalence of ICEHo in the *M. hominis* strains, the four CDSs that are part of the minimal ICE backbone (CDS1, CDS5, CDS17, and CDS22) and the specific *M. hominis* ICE module were searched by PCR in a panel of 120 *M. hominis* clinical isolates (Table 6). A complete ICEHo was considered present when the four CDSs and the specific *M. hominis* ICE module were detected. The data showed that 45% of the isolates possessed the four CDSs and the specific *M. hominis* ICE module, suggesting the presence of complete ICEHos. In 35% of isolates, none of the four CDSs or the specific *M. hominis* ICE module were detected, suggesting the absence of ICEHo. The remaining 20% of clinical isolates harbored one, two or three CDSs, suggesting the presence of potential ICEHo remnants (Table 6). The specific *M. hominis* ICE module was present in 54.5% (6/11) of strains harboring three backbone CDSs. Among the 27 *M. hominis* isolates carrying the *tet*(M) gene, the prevalence of the four CDSs and the specific *M. hominis* module was 48.1% (13/27), whereas among the 93 *M. hominis* isolates not carrying the *tet*(M) gene, the prevalence of the four CDSs and the specific *M. hominis* module was 44.1% (41/93). There was no significant difference in the prevalence of ICEHos between isolates carrying the *tet*(M) gene and those without the *tet*(M) gene (*P* = 0.85, χ^2^ test).

**TABLE 6:** Distribution of the four ICE backbone CDSs and the specific *M. hominis* ICE module in 120 clinical isolates of *M. hominis.*

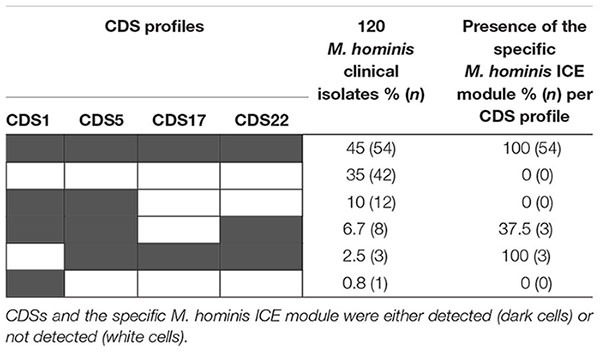			

## Discussion

Since their discovery, MICEs have been found distributed among several mycoplasma species (Calcutt et al., 2002; Marenda et al., 2006; Thiaucourt et al., 2011; Wise et al., 2011; Tardy et al., 2015) and there is accumulating evidence that they play a pivotal role in HGTs of large DNA fragments (Dordet Frisoni et al., 2013; Dordet-Frisoni et al., 2014; Citti et al., 2018). This report provides the first description of ICE in *M. hominis* and highlights their specific and new features.

The previously reported MICE backbone, comprised of CDS1, CDS5, CDS17, and CDS22, was found (Tardy et al., 2015) in *M. hominis* ICEs. These four CDSs were demonstrated to be part of the minimal ICE machinery using a transposon-based strategy in *M. agalactiae* (Baranowski et al., 2018). The gene repertoire and organization of CDSs in ICEHo were similar to those reported in *M. agalactiae* (Marenda et al., 2006) and *M. fermentans* (Calcutt et al., 2002), two other species belonging to the Hominis phylogenetic group. Thus, ICEHos also belong to the previously designated “hominis” MICE type (Tardy et al., 2015). Notably, five CDSs (CDS5, CDS15, CDS16, CDS17, and CDS19) harboring transmembrane domains and previously described as candidates for conjugative channel formation (Citti et al., 2018), were identified in ICEHos. CDS14, a surface-exposed lipoprotein essential for mycoplasma conjugation and predicted to initiate cell-to-cell contact in the first step of conjugation (Baranowski et al., 2018; Citti et al., 2018), was also identified in ICEHos as well as CDS22, encoding the DDE recombinase essential for ICE excision and integration (Baranowski et al., 2018; Citti et al., 2018). These observations raised questions regarding the functionality of ICEHos. As the first step in conjugative ICE transfer is excision from the chromosome, we searched for circular extrachromosomal forms and these were found in five of the seven strains harboring ICEHos. Interestingly, the two ICEHos for which no circular forms were found had truncated ends, i.e., the 3′ end of CDS1 in ICEHo 4016 and the 5′ end of CDS22 in ICEHo 5012 (Supplementary Figure S1). The absence of IRs at both extremities of ICEHo 5012 and ICEHo 4016 was also noted, suggesting that both complete 3′ and 5′ ends of the ICEHo are required for excision from the chromosome. In contrast, the 3′ end of ICEA CDS1 was shown to be non-essential in *M. agalactiae*, as miniICEs composed of IRs and the first 470 nt of CDS1 were able to circularize (Dordet Frisoni et al., 2013). Mating experiments are needed to confirm the capacity of ICEHos to transfer. However, the transformation of *M. hominis* is problematic (Bébéar et al., personal data) and to date no *M. hominis* transformants harboring an ICEHo marked by an antibiotic resistance gene marker have been obtained, thus limiting the possibility of performing such experiments.

As previously reported in MICE (Citti et al., 2018), the present study showed that *M. hominis* strains can carry a single or two similar ICEHo copies. The insertion sites of ICEHo in the chromosome were variable and could occur within CDSs, suggesting that the DDE recombinase encoded by CDS22 has no specific target site and that random insertion may sometimes have deleterious effects. The prevalence of ICEHo in *M. hominis* strains was high, i.e., 45% in a collection of 120 *M. hominis* clinical isolates. This prevalence may have been slightly underestimated because six additional strains (5%) harbored the specific *M. hominis* ICE module and three of the four backbone CDSs. We cannot exclude the possibility that the fourth CDS would not have been detected by PCR due to slight modifications of the gene sequence. In addition, CDS1, CDS5, CDS17, CDS22 and the specific *M. hominis* ICE module are also present in two previously published genome datasets for two other clinical isolates, *M. hominis* PL5 (GenBank JRXA01000000) and *M. hominis* TO0613 (GenBank CP033021). Overall, this 45% prevalence was equivalent to that of 47% (78/166) found in a collection of four ruminant mycoplasma species (Tardy et al., 2015). It should also be noted that CDS22 from ICEHo 4788 has a match in the genome of *U. parvum* serovar 14 (ATCC 33697), another human mycoplasma sharing the same genital niche. Thus, ICEs appear to be widespread both within animal and human mycoplasmas. The maintenance of such genetic mobile elements in genomes, as small as those of mycoplasmas, supports the hypothesis that MICEs may confer a positive advantage for the physiology or pathogenicity of the bacterium. Nevertheless, the prevalence of ICEs was not higher in isolates carrying the *tet*(M) gene responsible for tetracycline resistance. This suggests that *tet*(M)-mediated tetracycline resistance is not attributable to ICEHos in *M.* hominis. Indeed, in previously sequenced strains harboring the *tet*(M) gene, the resistance gene was carried by a transposon that integrated into the *rumA* gene in a site-specific manner (Allen-Daniels et al., 2015; Calcutt and Foecking, 2015a; Chalker et al., 2018), which is not a gene carried by *M. hominis* ICEs. However, when focusing on isolates harboring no ICE-specific CDSs in their genome, it was noted that 43% (40/93) were *tet*(M)-negative strains, whereas only 7.4% (2/27) were *tet*(M)-positive strains (*P* < 0.01). This finding suggests a possible association between the absence of ICE and the absence of the *tet*(M) gene in the genome. Therefore, we speculated that *M. hominis* strains that do not carry ICEs may be less susceptible than others to the entry of foreign DNA, such as the *tet*(M) gene, by HGT. The reason for this phenomenon is unknown, but it will be of interest to screen for differences in the nature and number of restriction-modification systems between such strains.

The major difference with previously reported MICEs was the presence of a specific *M. hominis* ICE module, not found in other MICEs, composed of five to six juxtaposed CDSs that had no orthologs with any MICE CDSs, and no predicted transmembrane domains. Interestingly, Phyre2 3-D structure prediction indicated that two proteins, MhoF and MhoH, share common structural features with TAL effectors, found in *Xanthomonas* spp. and in *Ralstonia solanacearum* (Heuer et al., 2007; de Lange et al., 2013), and with BurrH from *Burkholderia rhizoxinica* (Juillerat et al., 2014; Stella et al., 2014). TAL effectors are heterogeneous transcription factors involved in polynucleotide recognition and signal transduction, and are delivered into plant cells by pathogenic bacteria *via* a type III secretion system (Doyle et al., 2013; Pereira et al., 2014). Many TAL effectors are important virulence factors (Schornack et al., 2006; Boch and Bonas, 2010; Mak et al., 2013). The results of *in silico* analysis indicated that *mhoF* and *mhoH* are phylogenetically related and belong to the same multigene family. They most likely appeared by duplication and their sequences have diverged gradually during evolution.

Our study raised the question of whether MhoF and MhoH retained DNA-binding capability similar to BurrH, which also lacks typical TAL effector features other than repeats (Juillerat et al., 2014; Stella et al., 2014). Considering the predicted structural elements, DNA-binding properties of MhoH and more especially of the larger MhoF cannot be excluded. Indeed, MhoF has a double-helix structure similar to the minimal functional TAL effectors (Boch et al., 2009) (Figure 3A). The identified putative RVDs in MhoF and MhoH sequences include the di-residues KG, FN, NG, HG, and QG. Extensive studies using natural or artificial RVDs showed that NG, HG, QG, and KG specifically or preferentially bind thymine, while FN preferentially binds guanine (Boch et al., 2009; Yang et al., 2014; Miller et al., 2015). However, the genes encoding the bacterial apparatus, which would be required if these proteins were secreted, have not yet been identified in *M. hominis*. Nevertheless, *M. hominis* ICEs encode a putative type IV secretion system (T4SS). Although to date TAL effectors have only been associated with type III secretion systems, it remains possible that *M. hominis* ICE repeat-containing proteins could be transferred through this T4SS, either as free proteins or as proteins bound to DNA. Indeed, T4SS were shown to translocate proteins (effectors), DNA or DNA–protein complexes (Grohmann et al., 2018). Further studies are therefore needed to determine whether MhoF and MhoH retain DNA-binding capacity and can be transferred through a T4SS, or if another secretion system is involved. In addition, as some TAL RVDs are involved in the recognition of modified nucleotides (Deng et al., 2012), MhoF and MhoH may act together with other ICEHo proteins, such as MhoI and MhoK, which are predicted methyltransferases. The presence of such systems in *M. hominis* may be relevant for its survival, pathogenicity and/or virulence. Interestingly, *M. hominis* was shown to undergo endosymbiosis with *Trichomonas vaginalis*, a human urogenital pathogenic protozoa responsible for sexually transmitted infections. This symbiosis was observed in up to 94% of clinical isolates of *T. vaginalis* (Rappelli et al., 1998; Furnkranz et al., 2018), and it was recently reported that *M. hominis* has an impact on the gene expression of *T. vaginalis* (Furnkranz et al., 2018). As TAL effectors were only reported in bacteria able to live in symbiosis with other organisms, we speculate that ICEHos harboring TAL-like effectors may provide favorable properties to *M. hominis* and/or *T. vaginalis*, to enable survival in their environment.

In addition, a conserved motif of 23 aa localized within intrinsically disordered domains (IDRs) was found in several proteins of ICEHo. IDRs are involved in essential cell processes through two basic mechanisms, i.e., the entropic chain mechanism responsible for rapid fluctuations among many alternative conformations and molecular recognition via short recognition elements (Mitic et al., 2018). They were previously described in *M. pneumoniae* (Miyata and Hamaguchi, 2016) and *M. genitalium* (Dunker et al., 2000). It is not surprising to find IDRs in mobile elements, such as ICEHos, because plasmid-encoded proteins were shown to contain considerably more IDRs than chromosome-encoded proteins (Mitic et al., 2018). This motif was only found in a hypothetical protein of *Ca. Mycoplasma girerdii* (MGM1_3750). *Ca. M. girerdii* is an uncultivated urogenital mycoplasma recently identified in vaginal samples by a metagenomic strategy (Martin et al., 2013). As per *M. hominis*, *Ca. M. girerdii* is tightly associated with *T. vaginalis* (Fettweis et al., 2014). Notably, *T. vaginalis*, *Ca. M. girerdii*, and *M. hominis* are able to co-localize in the vaginal tract (Fettweis et al., 2014), suggesting the possibility of HGT between the two urogenital mycoplasma species.

## Conclusion

This study described the presence of ICEs in almost half of all *M. hominis* clinical isolates and these MGEs are unlikely to be associated with the antibiotic resistance *tet*(M) gene. All *M. hominis* ICEs include a specific *M. hominis* module harboring proteins that share common structural features with TAL effectors involved in polynucleotide recognition and signal transduction in symbiotic bacteria. This type of ICE may confer a selective advantage on the bacteria to allow survival in their environment, or transformation to pathogenic status, but further experiments are needed to check their functionality in the *M. hominis* species.

## Data Availability Statement

The datasets generated for this study can be found in the SRA BioProject PRJNA493181, GenBank CP035542, CP038014, and CP035543.

## Author Contributions

SP and CB conceived and designed the study. AM, OP, and LB performed the research. AM, OP, ED-F, PS-P, CC, LB, and SP analyzed the data. AM, LB, and SP wrote the manuscript. ED-F, PS-P, CC, OP, and CB critically revised the manuscript.

## Conflict of Interest

The authors declare that the research was conducted in the absence of any commercial or financial relationships that could be construed as a potential conflict of interest.

## Footnotes

1 http://www.clcbio.com/products/clc-assembly-cell

2 https://github.com/rrwick/Filtlong

3 https://blast.ncbi.nlm.nih.gov

4 www.ebi.ac.uk/interpro/search/sequence-search

5 http://services.cbib.u-bordeaux.fr/molligen/

6 https://www.sanger.ac.uk/science/tools/artemis-comparison-tool-act

7 https://www.ebi.ac.uk/Tools/psa/emboss_needle/

8 http://www.cbs.dtu.dk/services/TMHMM/

9 http://meme-suite.org/tools/meme

10 https://www.ebi.ac.uk/Tools/pfa/radar/

11 http://sparks-lab.org/SPINE-D/

12 http://www.sbg.bio.ic.ac.uk/phyre2/html/page.cgi?id=index

13 http://bioinfo.protres.ru/IsUnstruct/

14 http://www.pondr.com/
