# Supplementary material for: High Prevalence of Integrative and Conjugative Elements Encoding Transcription Activator-Like Effector Repeats in Mycoplasma hominis
Source: Front Microbiol. 2019 Oct 18;10:2385. doi: 10.3389/fmicb.2019.02385 (PMC6813540; doi:10.3389/fmicb.2019.02385)
Supplement: Supplementary file 1 [file Image_1.pdf]

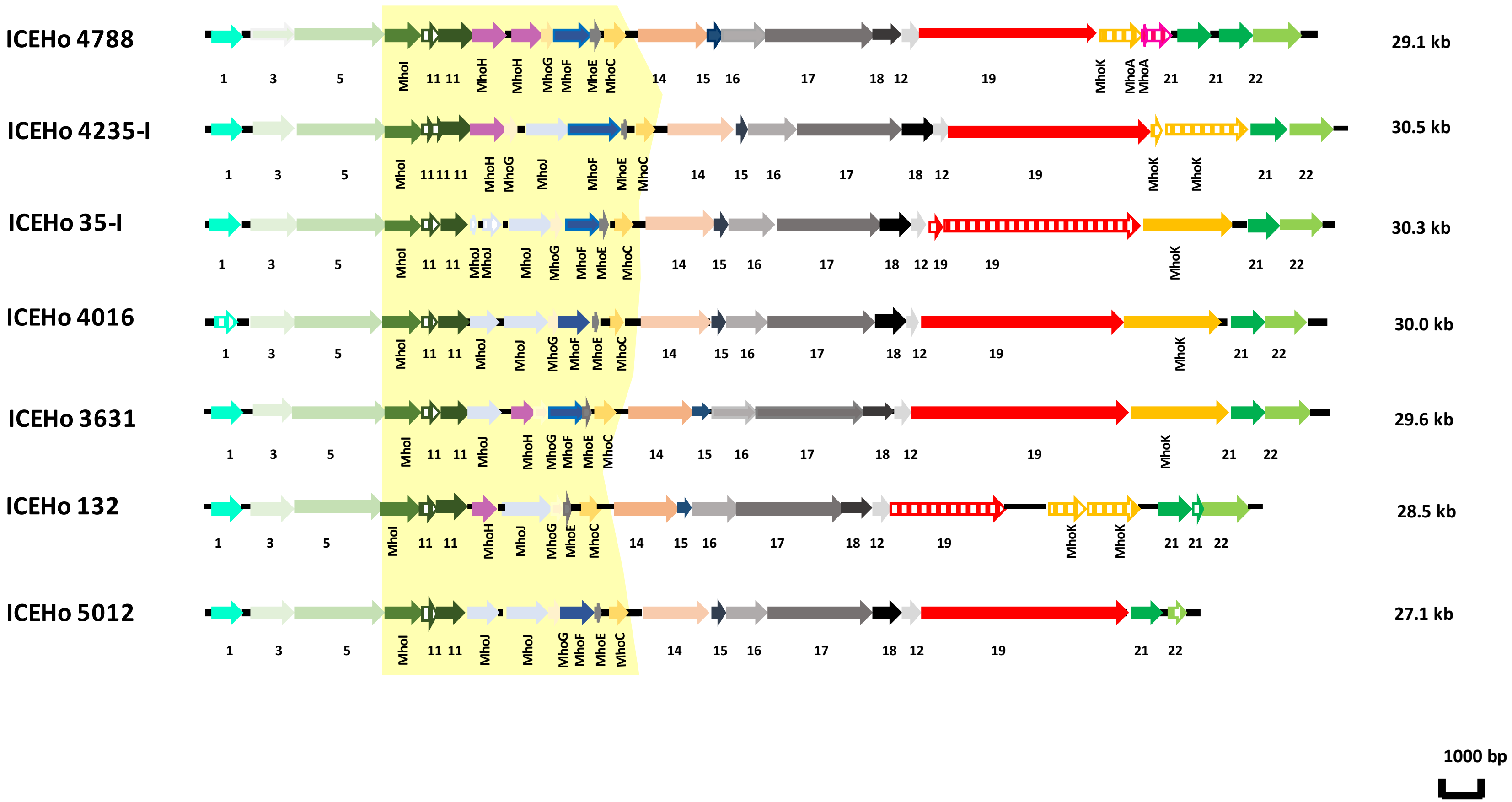

Figure S1. Genetic organisation of ICEHos in seven fully sequenced strains of *M. hominis*.

Homologous CDSs are indicated by the same color and the same numbers using the previously described nomenclature of ICE CDSs. MhoA, MhoC, MhoD, MhoE, MhoF, MhoG, MhoH, MhoI, MhoJ, and MhoK are *M. hominis* ICE CDSs that have not been reported in other MICEs. CDSs harboring chopped color represent pseudogenes.
