# Supplementary material for: High Prevalence of Integrative and Conjugative Elements Encoding Transcription Activator-Like Effector Repeats in Mycoplasma hominis
Source: Front Microbiol. 2019 Oct 18;10:2385. doi: 10.3389/fmicb.2019.02385 (PMC6813540; doi:10.3389/fmicb.2019.02385)
Supplement: Supplementary file 2 [file Image_2.pdf]

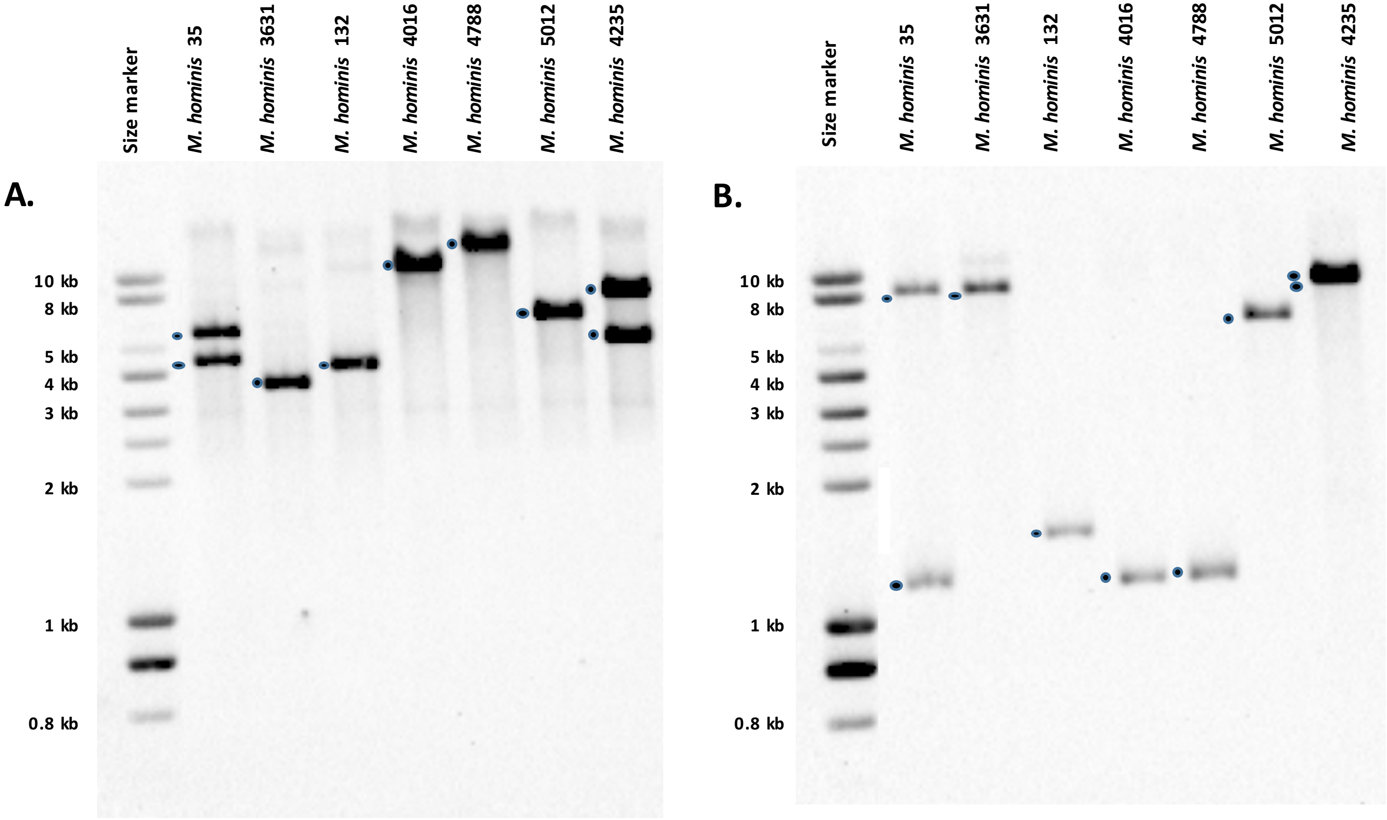

Figure S2. Southern blotting analysis of the seven fully sequenced *M. hominis* strains harboring complete ICEHos.

(A) Genomic DNA digested with *Eco*RV and probed with digoxigenin-labelled CDS1 gene probe.

(B) Genomic DNA digested with *Eco*RV and probed with digoxigenin-labelled CDS22 gene probe. Dots indicate the fragment displaying the expected size and corresponding to the ICEHs described in this study.
