# Supplementary material for: High Prevalence of Integrative and Conjugative Elements Encoding Transcription Activator-Like Effector Repeats in Mycoplasma hominis
Source: Front Microbiol. 2019 Oct 18;10:2385. doi: 10.3389/fmicb.2019.02385 (PMC6813540; doi:10.3389/fmicb.2019.02385)
Supplement: Supplementary file 3 [file Image_3.pdf]

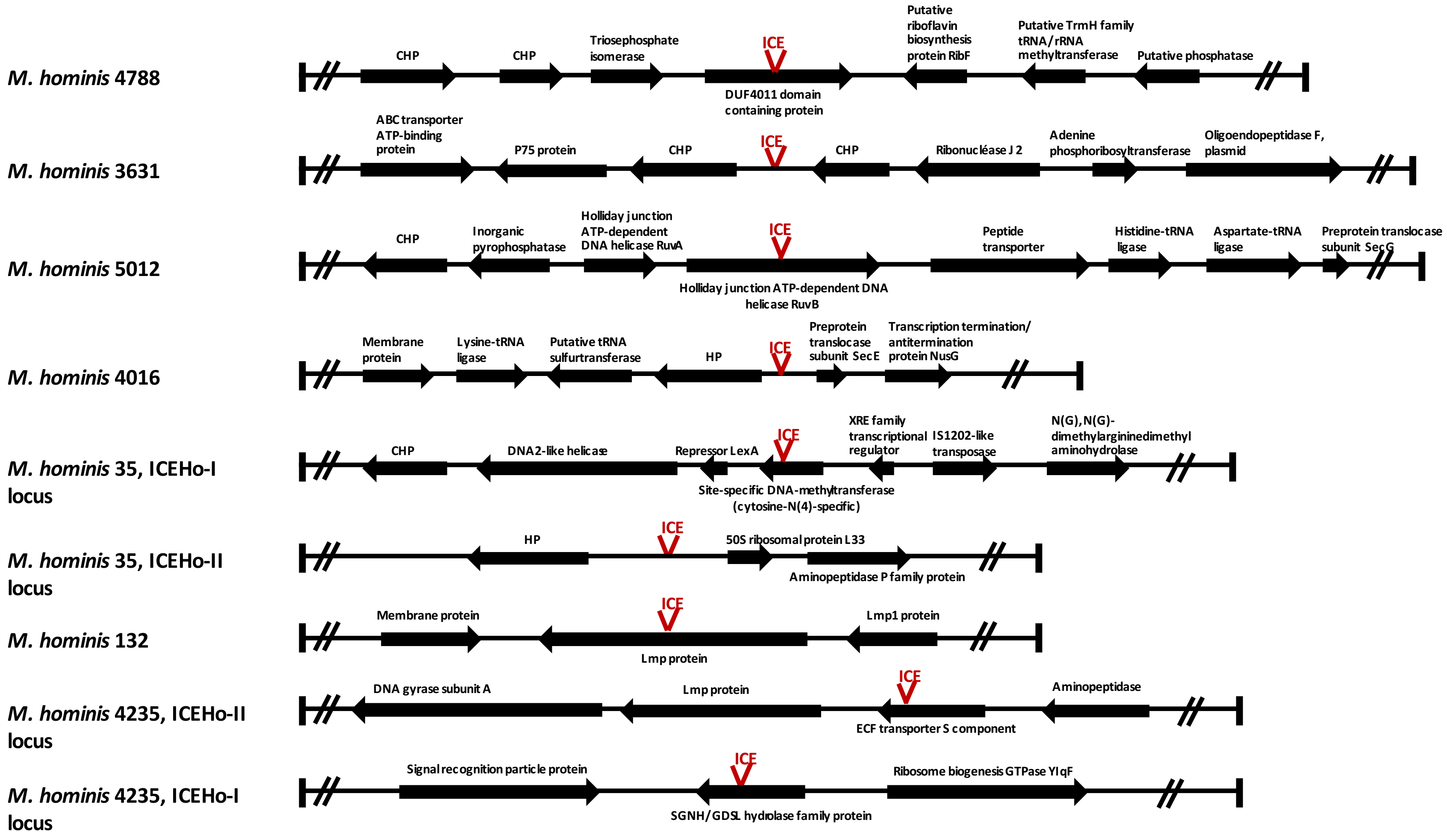

Figure S3: Genomic locations of ICEH insertion sites in the seven *M. hominis* strains harboring complete ICEHos.

HP, hypothetical protein; CHP, conserved hypothetical protein.
