# Supplementary material for: High Prevalence of Integrative and Conjugative Elements Encoding Transcription Activator-Like Effector Repeats in Mycoplasma hominis
Source: Front Microbiol. 2019 Oct 18;10:2385. doi: 10.3389/fmicb.2019.02385 (PMC6813540; doi:10.3389/fmicb.2019.02385)
Supplement: Supplementary file 4 [file Image_4.pdf]

```
1       MKIKAFNRDQ IEQIWLGLNS GLDVSIYAKQ NNFNRPIYDC LQMAQIRWGL

51      EAGLDVSVYA KQNNFNRPIF NWSQMEQIRK GLKQGLDVSQ YTQLDHKGHP
                                                    *
101     IYDGNQMSQI REGLVQGLDV TQYTQLDHKG HPIYDWFQMA QIRYDLEASL
         *                      *
151     DISQYTQLDL KGHPIYNGYQ MNQIWWGLKN GLDISQYNKL DPNGHPIYDW

201     AQMEQIRIGL EKGLDISKYN QLDPHGHPIF DNNQMEQIRW GLGKSLDISQ
                           *
251     YTQLDHKGRP IYDWWQMEQI RKGLVQGLDV SKHTHLASKS SLSDFDKTPT
        *                                         * * *    * *
301     DLKDSKVINE YNKSSHTPSD TQSKSRKL
            *      *   *   *  * * *
```

Figure S4: Prediction of disordered domains and phosphorylated serine, threonine, and tyrosine residues in MhoF from ICEH 4788.

Prediction of disordered domains was performed using IsUnstruct, and prediction of phosphorylated serine, threonine, and tyrosine residues using DEPP server. Stars indicate predicted phosphorylation sites. Amino acids in red represent predicted disordered domains. The conserved 23-aa sequence is shown in bold.
