# Supplementary material for: High Prevalence of Integrative and Conjugative Elements Encoding Transcription Activator-Like Effector Repeats in Mycoplasma hominis
Source: Front Microbiol. 2019 Oct 18;10:2385. doi: 10.3389/fmicb.2019.02385 (PMC6813540; doi:10.3389/fmicb.2019.02385)
Supplement: Supplementary file 5 [file Table_1.pdf]

Table S1. Oligonucleotides used for the detection of extrachromosomal ICE circular forms

**Forward primers**	**Sequences of forward primers (5′-3′)**	**Reverse primers**	**Sequences of reverse primers (5′-3′)**
35-F-circu	GAATGGTTGGATTTCACTCTGG	35-R-circu	AGCTGTGTGTTTGGCTCTTC
4016-F-circu	CCCTTGTTTGCTAATGCTACTC	4016-R-circu	GCTGGGAGCCATTTATTACTG
5012-F-circu	GAATGGTTGGATTTCACTCTGG	5012-R-circu	TTAGCCTCTTGCTGCATTACC
132-F-circu	TCCCTTGTTTGCTAATGCTACG	132-R-circu	TTTAGCCTCTTGCTGCATTACC
4788-F-circu	GAATGGTTGGATTTCACTCTGG	4788-R-circu	GCATCCACAAGGCTATAATCG
3631-F-circu	TTCCGTGGTTCTTCCTTTGC	3631-R-circu	TAGCCTCTTGCTGCATTACC
4235-F-circu	CTTTGCCCTATTCAAGGGTG	4235-R-circu	TAGCCTCTTGCTGCATTACC
