# Supplementary material for: High Prevalence of Integrative and Conjugative Elements Encoding Transcription Activator-Like Effector Repeats in Mycoplasma hominis
Source: Front Microbiol. 2019 Oct 18;10:2385. doi: 10.3389/fmicb.2019.02385 (PMC6813540; doi:10.3389/fmicb.2019.02385)
Supplement: Supplementary file 6 [file Table_2.pdf]

Table S2. Comparison of CDSs from *M. hominis* ICEHo 4788 with CDSs from *M. fermentans* PG18 ICEF-II and *M. agalactiae* 5632 ICEA-III using BLAST.

ICEHo 4788		*M. fermentans* PG18 ICEF-II				*M. agalactiae* 5632 ICEA-III			
**CDS**	**Size (aa)**	**Size (aa) of PG18 ICEF-II CDSs**	**% coverage**	**% identity**	**E-value**	**Size (aa) of 5632 ICEA-III CDSs**	**% coverage**	**% identity**	**E-value**
CDS1	263	263	100	35.71	5e-38	264	71	30.99	3e-9
CDS3	413	400	98	37.56	1e-87	none	none	none	none
CDS5	766	740	64	44.75	3e-135	670	69	42.11	3e-130
CDS11	252	238	78	32.67	6e-25	221	9	30.43	0.37
CDS14	562	552	82	23.75	5e-19	525	97	21.63	1e-13
CDS15	120	95	45	44.74	1e-9	121	76	32.29	1e-14
CDS16	400	396	90	29.49	2e-36	357	67	25.68	1e-18
CDS17	927	937	98	40.04	0	928	99	40.26	0
CDS18	260	227	93	34.66	2e-39	none	none	none	none
CDS12	130	183	91	40.50	7e-24	134	65	31.03	3e-8
CDS19	1434	1424	73	31.83	2e-134	1517	76	30.97	5e-81
CDS21	286	313	88	68.50	6e-110	none	none	none	none
CDS22	396	390	77	34.98	1e-28	378	80	32.96	2e-34
